# New Insights into the Role of Neuron-Specific Enolase in Neuro-Inflammation, Neurodegeneration, and Neuroprotection

**DOI:** 10.3390/brainsci8020033

**Published:** 2018-02-18

**Authors:** Azizul Haque, Rachel Polcyn, Denise Matzelle, Naren L. Banik

**Affiliations:** 1 Department of Microbiology and Immunology, Medical University of South Carolina, Charleston, SC 29401, USA; polcyn@musc.edu (R.P.); baniknl@musc.edu (N.L.B.); 2 Department of Neurosurgery, Medical University of South Carolina, Charleston, SC 29401, USA; matzeldd@musc.edu; 3 Ralph H. Johnson Veterans Administration Medical Center, Charleston, SC 29401, USA

**Keywords:** neuron specific enolase (NSE), spinal cord injury (SCI), gliosis, cathepsin X, degeneration, neuroprotection

## Abstract

Neurodegeneration is a complex process that leads to irreversible neuronal damage and death in spinal cord injury (SCI) and various neurodegenerative diseases, which are serious, debilitating conditions. Despite exhaustive research, the cause of neuronal damage in these degenerative disorders is not completely understood. Elevation of cell surface α-enolase activates various inflammatory pathways, including the production of pro-inflammatory cytokines, chemokines, and some growth factors that are detrimental to neuronal cells. While α-enolase is present in all neurological tissues, it can also be converted to neuron specific enolase (NSE). NSE is a glycolytic enzyme found in neuronal and neuroendocrine tissues that may play a dual role in promoting both neuroinflammation and neuroprotection in SCI and other neurodegenerative events. Elevated NSE can promote ECM degradation, inflammatory glial cell proliferation, and actin remodeling, thereby affecting migration of activated macrophages and microglia to the injury site and promoting neuronal cell death. Thus, NSE could be a reliable, quantitative, and specific marker of neuronal injury. Depending on the injury, disease, and microenvironment, NSE may also show neurotrophic function as it controls neuronal survival, differentiation, and neurite regeneration via activation of phosphatidylinositol-4,5-bisphosphate 3-kinase (PI3K) and mitogen-activated protein kinase (MAPK) signaling pathways. This review discusses possible implications of NSE expression and activity in neuroinflammation, neurodegeneration, and neuroprotection in SCI and various neurodegenerative diseases for prognostic and therapeutic potential.

## 1. Introduction

Enolase is a glycolytic enzyme that catalyzes the conversion of 2-phosphoglycerate to phosphoenolpyruvate. There are three isozymes of enolase: enolase α is ubiquitous, enolase β is muscle-specific, and enolase γ is neuron-specific [1,2]. Neuron specific enolase (NSE) is a marker for neurons and peripheral neuroendocrine cells that exist as either γγ or αγ dimeric isozymes. While the γγ form of NSE is expressed in neurons, the αγ form is expressed in microglia, oligodendrocytes, and astrocytes [3,4,5]. This localization in neuronal and glial cells indicates that NSE may exert both inflammatory and neurotrophic activity regulating neuronal growth, differentiation, survival, and death.

Early studies suggest that NSE, which is measurable in blood and cerebrospinal fluid, could be a potentially useful biomarker for assessing neuronal damage and the prognosis of brain injury [6,7,8,9,10]. Higher concentrations of NSE have been detected in the gray matter of adult brains, while lower levels of NSE are reported in the white matter. Thus, NSE could be a biochemical marker to estimate neuronal damage in brain lesions [11]. NSE has also been measured in cerebrospinal fluid (CSF) of rats after intrastriatal injection of kainic acid (KA), a glutamic kainate receptor agonist that induces excitotoxic neuron death at high concentrations [12]. KA is the most common neurotoxin used to produce neuro-pathologic changes seen in patients with temporal lobe epilepsy. The systemic or intracerebral administration of KA initially induces status epilepticus, which is followed by long-term changes such as spontaneous seizures and progressive neuronal damage, primarily in the hippocampus. Steinberg et al., detected a significant decrease in NSE striatal content following KA injection [12], suggesting that measurement of NSE in CSF could be a sensitive index of neuronal damage. Interactions of NSE with many other nuclear, cytoplasmic, or membrane molecules in the CNS also raise the possibility that NSE is associated with glial activation and neuronal damage [13,14,15]. 

NSE has been implicated in ischemia, hypoxia, and diverse metabolic, proliferative, inflammatory, autoimmune, and neurodegenerative diseases [16,17,18,19,20]. A gradual loss of neurons and synaptic connections are common features of neurodegenerative disorders [21], where the degree of neuronal loss correlates with increased levels of NSE in serum and CSF and clinical progression of the disease. Our recent study suggests an inflammatory role for NSE in acute spinal cord injury (SCI), a devastating and debilitating condition with progressive pathological changes that include complex and evolving molecular cascades [22,23,24]. The expression levels of NSE in different tissues following SCI in rats were found to be significantly increased after acute SCI and inhibition of NSE expression and activity decreased secondary injury in SCI [24]. Moreover, enolase/NSE inhibition has been shown to decrease inflammatory chemokines/cytokines, inhibit MMP-9 activation, modulate metabolic hormones, and reduce gliosis following SCI through distinct cellular and metabolic pathways [20,24]. However, the specific role of NSE in inflammatory conditions following SCI and the mechanisms by which high levels of NSE may influence neurodegeneration in SCI, remain unclear. 

While NSE is thought to be an important molecule that directly assesses damage to neurons, it may also be involved in neuronal repair. NSE has been shown to control neuronal survival, differentiation, and neurite regeneration by activating the PI3K/Akt and MAPK/ERK signaling pathways [18,25]. NSE-mediated activation of both PI3K and ERK1/2 pathways is required for neurite outgrowth, which may be attenuated by inhibition of MEK (MAPK/ERK kinase) and PI3K. This neurotrophic activity could be regulated by the cysteine protease, cathepsin X (Cat X), which cleaves the C-terminal end of the molecule and impairs its activity [26]. Interestingly, NSE-mediated PI3K activation also regulates RhoA kinase, a key regulator of actin cytoskeleton organization, and may influence both neurodegeneration and neuroprotection depending on the signal strength. Pairing an improved understanding of these processes with novel and existing tools to enable effective regulation of NSE in neurons and glia might provide new opportunities for treatment of central nervous system (CNS) injury.

## 2. NSE in Neurons and Glia

NSE is a glycolytic isoenzyme located in central and peripheral neurons and neuroendocrine cells. While neurons predominantly contain the γγ form of NSE, glial cells possess both the αγ form of NSE and non-neuronal enolase (NNE, α-enolase). NSE levels are thought to be low in embryonic brain and may increase with the morphological and functional maturation of neurons [27]. Conversely, NNE levels are high in embryonic brain and decrease with the maturation of neurons and increase in NSE content [27,28]. A switch from NNE to NSE may occur during progressive maturation, and some migrating neuronal cells may contain hybrid enolase (αγ), resulting from an incomplete switch over to NSE-containing cells [29]. The mature rat brain contains NSE, a hybrid form (neuronal), and a non-neuronal enolase (glial form). Decreased levels of γγ NSE and αγ NSE (hybrid form) have been found following neuronal damage with no change in the activity of non-neuronal enolase. 

In addition to its localization in neurons, NSE has been found in several types of glial cells. Recent studies suggest that NSE is expressed in microglia [5,30], which play a differential role in neurodegeneration and neuroprotection. Microglial activation in the CNS is thought to be heterogeneous where two so-called phenotypes, M1 and M2, can produce cytotoxic and neuroprotective effects, respectively [31]. Whether M1 type microglia express higher levels of NSE as compared to M2 type microglia remains unclear, but the potential connection between NSE and microglia-related differentiation is discussed further in later sections of this review. NSE is also expressed in oligodendroglial cells at various stages of their differentiation and maturation, but it is repressed in adult fully mature cells [32]. NSE is expressed in astrocytes, especially in reactive astrocytes undergoing various morphological changes [33]. The differential expression of NSE may be related to degenerative, neurotrophic or neuroprotective functions of astrocytes in neuronal injury, for reactive astrocytes can contribute to the death of neurons and oligodendrocytes in neurodegenerative disorders [34,35,36,37]. 

Studies have examined the regulation of NSE expression in oligodendrocytes at various steps of their differentiation and maturation and have shown that NSE is expressed in oligodendroglial cells in vitro and in vivo [32,38]. Investigation of the role NSE plays in oligodendrocyte maturation and remyelination after CNS injury would be interesting. While heterogeneity of NSE expression was observed in cultured oligodendrocytes and adult rat brain, no significant change in other enolase expression was detected. This is striking because α-enolase can typically be converted into γ-enolase (NSE) in the brain. These studies have also shown that NSE gene expression, associated with the differentiation of oligodendrocytes, is repressed in adult fully mature cells.

## 3. NSE as Prognostic Factors in Patients during Neuroinflammation

**Figure 1:**
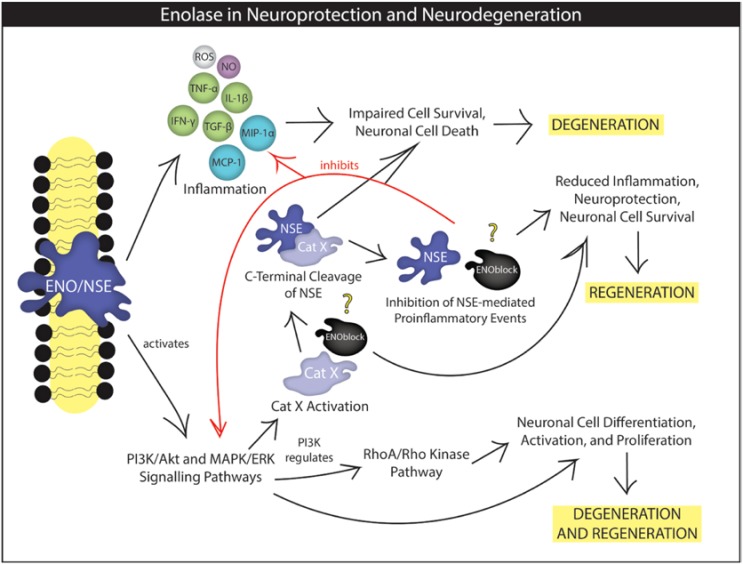
Elevation of enolase promotes glycolysis, cellular proliferation, activation and migration via the PI3K/AKT and MAPK/ERK pathways. Enolase-mediated activation of PI3K also regulates RhoA kinase, which influences actin cytoskeleton reorganization, induction of neurite outgrowth, and growth arrest in neuronal cells. NSE is a substrate of Cat X, and regulation of NSE by Cat X may determine neuronal survival during CNS injury. Treatment with enolase inhibitor, ENOblock, may attenuate NSE, Cat X, and other inflammatory events in SCI, thus reducing neuroinflammation and neurodegeneration and promoting neuronal survival and/or neuroprotection.

Inflammation is a major contributor to the pathophysiology of heart attacks, strokes, kidney problems, neurological deficits, and many other complications [39,40,41,42]. In these conditions, the inflammatory agents in the environment and cellular receptors, such as cell surface enolase in SCI, are important determinants of inflammatory activation (Figure 1). Cells may undergo various stress responses upon cell surface enolase expression, which can promote multiple cellular activation pathways including cytokine- and chemokine-derived activation of inflammatory responses. In many cases, the inflammation is mediated by activated microglia and reactive astrocytes which release inflammatory mediators to the neuronal microenvironment, potentially determining neural survival and death. 

While the biological half-life of NSE in body fluids is about 24h, elevated levels of NSE in serum and/or CSF may trigger activation of different cellular pathways leading to neuroinflammation. Homeostatic states within cells can also contribute to inflammatory activation through modulation of NSE-mediated pro-survival or death pathways. Our group has shown that enolase/NSE inhibition decreases inflammatory chemokines/cytokines, inhibits MMP-9 activation, modulates metabolic hormones, and reduces gliosis following SCI [20,24]. NSE-mediated activation of PI3K and MAPK pathways may promote inflammatory cytokines and chemokines, which facilitate neurodegeneration (Figure 1). This activation also regulates the RhoA/Rho kinase pathway in brain injury to promote inflammation and neuronal death [18]. In this scenario, inhibition of RhoA could result in enhanced NSE-induced neurite outgrowth, accompanied by actin polymerization and its redistribution to growth cones. Activation of PI3K-Akt and MAPK-ERK signaling occurs by the extracellular stimulation (e.g., alpha enolase) which may be associated with the inhibition of neuronal cell death. PI3K-Akt signaling is known to play a pro-survival and regenerative role in neurons, but could also contribute to gliosis and negative aspects of the lesion and surrounding tissue environment Overexpression of Cat X is detrimental to NSE and neuron because the minimum requirement of NSE for neural cell survival is lost as a result of the catalytic activity of Cat X. While complete inhibition of NSE is not desired, attenuation of NSE expression and activity in injured tissues (e.g., SCI) by a pharmacological inhibitor could be beneficial. In this scenario, a combination approach targeting Cat X and NSE could be designed to balance inhibiting pro-death aspects of NSE and supporting regenerative and pro-survival aspects. 

While its specific role in inflammation is still being fully elucidated, NSE is considered a biomarker of neuronal stress and has prognostic potential for a variety of neurological disorders. Serum NSE levels are significantly elevated in patients with unfavorable neurological outcome in a variety of conditions. Furthermore, the degree of disability and neurological deficits often correlate with increased NSE concentrations, indicating its prognostic value. Elevated NSE is thought to be a marker of oxidative damage and is the underlying parameter of several neurodegenerative disorders, including Huntington disease (HD), Friedreich ataxia, hereditary spastic paraplegia, rare familial forms Parkinson disease (PD), Alzheimer disease (AD), and amyotrophic lateral sclerosis (ALS) [43,44,45,46,47,48,49,50]. In a recent study, NSE serum concentrations were measured in cardiac arrest patients from five hospitals in Germany, Austria, and Italy, and the outcomes were analyzed [51]. High NSE serum concentrations predicted poor neurological outcome in a majority of cardiac arrest patients [52]. NSE levels have also been used to predict neurological outcome in traumatic brain injury (TBI) [53] and retinal disease, such as retinal detachment [54,55,56]. Higher levels of NSE have been detected in Leber’s hereditary optic neuropathy (LHON), a mitochondrial disease affecting retinal ganglion cells (RGC) [56]. Interestingly, a marked elevation of serum NSE levels were found in male LHON carriers, suggesting that male carriers could be at higher risk for LHON-related neuronal stress. Recent studies have shown that serum NSE has high predictive value for determining severity and early neurobehavioral outcome after acute stroke and other adverse neurologic events [57,58,59]. NSE serum values are found to be relatively low at the beginning of ischemic brain injury and may rise to a point considerably higher in patients with unfavorable outcomes than in patients with favorable outcomes even after 6 months [60,61]. Increased levels of NSE are also detected in diabetic subjects, and higher levels of NSE are closely related to diabetic neuropathy. NSE is aberrantly expressed in glioblastoma cell lines and patient biopsies, and knockdown of NSE has been shown to potentiate the effect of chemotherapy and radiotherapy with increased survival [62]. Thus, elevation of NSE should be actively considered in tumors, acute brain diseases, and hemolysis. 

In addition to evaluating serum for biomarkers of neural damage, a number of studies have aimed at validating a plasma biomarker for neuronal damage that can be used in acute and chronic models of neurological diseases. Plasma NSE is a valid and simple experimental biomarker that allows for quantification of the degree of neuronal injury in a non-invasive approach [63]. Higher plasma NSE concentrations were associated with mortality in critically ill septic patients, suggesting that NSE may have utility as a marker of neuronal injury in sepsis [64]. In EAE, plasma NSE levels have been shown to correlate with neurological scores and histopathological damage of axons at different time points. Elevated plasma NSE levels also correlated significantly with stroke size, EAE score and histopathological damage in EAE. Plasma levels of NSE are elevated in various conditions of CNS damage [65,66]. NSE is elevated in some types of cancer, head trauma, cerebral damage after cardiac surgery, and cerebral infarction [11,67,68]. Thus, NSE has diagnostic and prognostic potential for inflammation and neuronal damage in each of these conditions. Although the concentrations of NSE in serum and CSF have been used as a biomarker in injuries, cancers, and neurodegenerative diseases [49,69], NSE is also present in erythrocytes [70]. Therefore, careful analyses of hemolysis in pre-analytical conditions are needed before deciding whether or not to measure NSE levels for disease prediction.

## 4. NSE in Neuronal and Glial Cell Activation, Differentiation, and Migration

NSE expression and activity are frequently increased in neuronal and glial activation and injury, major factors implicated in neurodegenerative diseases [71,72]. In neuronal degeneration, glial cells regulate neuronal metabolism to support stressed neurons. Deficient mitochondrial metabolism in neuronal cells may generate reactive oxygen species (ROS) that enhance neuronal degeneration. In this regard, antioxidants may reduce the damaging effect of ROS, oxidative stress, and the extent of glial cell proliferation in the brain [73]. The distribution of activated glial cells and NSE expression and activity are important for controlling brain damage and improving function.

Microglia NSE expression and activity during glial differentiation and proliferation may indicate their activation status and responses to cellular damage [5,30]. Microglial activation in the CNS is heterogeneous because of the differentiation and expansion of M1 and M2 type microglial populations [31]. Microglia can produce either cytotoxic or neuroprotective effects depending on the phenotypes activated in the microenvironment. M1 type microglial populations are believed to be critically associated with the inflammatory neurodegenerative process whereas M2 microglia may take part in the repair process. Upon CNS injury, an increased expression of NSE in microglia, especially in M1 type microglia, may promote neurodegeneration. M1 microglial secreted factors are also neurotoxic and may induce neuronal cell death. Thus, regulation of M1/M2 microglia is important in attenuating inflammation and promoting neuroprotection. Based on this information, microglial and astroglial differentiation and their responses to various stimuli appear to be important parameters for designing targeted therapies against CNS injury.

Neurogenesis is thought to be more sensitive to SCI than astroglial and microglial responses, where NSE could play dual roles in neuronal survival and death (Figure 1). Microglial populations have been shown to proliferate and become activated in damaged neuronal tissue [31]. Activated and differentiated microglia may release various factors which modulate secondary neuronal degeneration and regeneration after SCI. In acute SCI, detrimental factors from activated M1 microglia are involved in the induction of damage in neurons and oligodendrocytes [5,30]. By contrast, differentiated M2 microglia show a neuroprotective effect via the production of neurotrophic factors. In this scenario, the role of NSE as well as other factors could be investigated in differential microglial activation and generation of both detrimental and beneficial effects as it remains unclear if NSE expression and activity modulate neuronal and glial migration and influence neural survival and death.

While activated microglia produce inflammatory cytokines and chemokines that may promote degeneration, microglia-derived cathepsin X (Cat X) can regulate NSE and neural fate (Figure 1). Recent studies have shown that inhibition of Cat X suppresses microglial activation via inhibition of the activity of MAPK and induces neuroprotection [5,30]. It would be interesting to examine whether inhibition of enolase decreases Cat X expression and activity and influences neuronal and glial activation, differentiation, and migration [26]. Neuronal and glial proliferation, differentiation, and migration are important factors for the formation of complete nervous system. The aforementioned changes could be detected by cellular markers and are valuable tools for examining the function of cells in normal conditions as well as during injury and disease.

## 5. NSE in Neurodegeneration and Neuroprotection

Microglial activation may also play dual roles in supporting neurodegeneration and neuroprotection, depending on the factors influencing microenvironment and the disease [74,75,76]. For example, amyloid β is a major component of the plaque deposits in Alzheimer’s disease that are considered a hallmark of the disease. Activated microglia are capable of phagocytosis of amyloid-β deposits [77,78] and of releasing neurotrophic factors (e.g., NGF, BDNF) that are neuroprotective [78,79]. Microglial phagocytosis of dead or dying neurons can also be beneficial by preventing the release of pro-inflammatory factors [80,81]. On the other hand, activated microglia can also phagocytose viable neurons, thus executing their death and inducing neurodegeneration [82,83]. In chronic inflammation, microglial activation can promote neurodegeneration, axonal damage, and neural death by the release of inflammatory cytokines and chemokines [84]. Axon degeneration is widespread both in neurodegenerative disease and in normal neural development, but the molecular pathways regulating these processes and the extent to which they are distinct or overlapping remain unclear. Molecular mechanisms that mediate neuronal death e.g., caspases and the Bcl-2 family members Bax and Bak, may be responsible for the induction of axonal damage and neural death.

New evidence suggests that NSE-mediated activation of degradation pathways with subsequent activation of inflammatory cytokines, chemokines, and other inflammatory mediators may initiate axonal damage [18,24,26]. Higher levels of NSE detected in SCI may be implicated in neuronal cell death in the gray matter. This increase in NSE may also be involved in the presence of reactive astrocytes and axonal degeneration in the dorsal, lateral, and ventral white-matter columns. Increased NSE expression and activity may also drive metabolic changes that take place in regions away from the epicenter in injured spinal cord. Secondary insult in SCI is characterized by destruction of neuronal and glial cells that leads to the expansion of the damage and loss of function [72]. Thus, mechanisms that either promote or prevent neuronal inflammation and cell death could be investigated to find new avenues for preventing and treating neurodegenerative disorders. An increased vulnerability to SCI may depend on the activation of NSE as well as a number of neuroprotective and neuro-destructive molecular signals in the damaged cord. Multiple factors, including inflammatory cytokines and chemokines, altered homeostasis of lipids and neurofilaments, and the failure of the damaged tissue to rein in oxidative damage and apoptotic cell death, are considered important in the induction of irreversible neurodegenerative processes.

It is possible that increased NSE expression and activity after SCI promote inflammatory events in spinal cord and activate degradative pathways leading to activation of inflammatory cytokines/chemokines, which aggravate secondary damages of SCI. Our recent study showed that NSE is markedly elevated after acute SCI and that inhibition of NSE by ENOblock decreased NSE levels in serum and spinal cord tissues, attenuated inflammatory cytokines and chemokines in serum, and reduced glial activation [24]. As some level of NSE is also required for neuronal survival, regulation of NSE expression and activity could be important for optimally controlling both NSE-mediated activation of inflammatory pathways and NSE-mediated neuronal cell survival. While this review discusses the role of NSE in glia activation and neuro-inflammation implicated in neurodegeneration, NSE is also involved in the energy-generating process of the cell. Under physiological conditions, reactive oxygen species (ROS) generated from mitochondria, are maintained at relatively low levels by endogenous antioxidants. Elevated NSE could alter the redox balance by promoting neural inflammation or abnormal mitochondrial function, which is tightly linked with neurodegenerative disorders.

NSE-mediated neurotrophic functions could be abolished by the proteolytic activity of Cat X, resulting in neurodegenerative disorders in the host [30]. As a cytoplasmic enzyme NSE is involved in increased aerobic glycolysis, supporting cellular proliferation. However, NSE at different cellular localization at pathophysiological conditions may participate in other cellular engagements (Figure 1). The C-terminal part of the molecule, which is not related to glycolytic pathway, is thought to control neuronal survival, differentiation, and neurite regeneration by activating phosphatidylinositol 3-kinase (PI3K) and mitogen-activated protein kinase (MAPK) signal transduction pathways [18]. A recent study has shown that NSE is a substrate of Cat X, which can proteolytically remove two C-terminal amino acids in NSE, impairing neuronal survival and neuritogenesis [5,85]. NSE’s neurotrophic activity is thus regulated by the proteolytic activity of the cysteine carboxypeptidase Cat X [85]. The interplay between NSE and Cat X under neuropathological conditions has been reported, where NSE rescued neuronal cells from degeneration caused by Aβ [5]. Specifically, neuronal-differentiated PC12 cells (rat adrenal phaeochromocytoma) were treated with Aβ and showed a significant dose-dependent decrease in neuronal cell survival. However, exposure to NSE resulted in almost complete preservation of cells, suggesting the importance of NSE in neural protection.

Damage to axons leads to neuronal cell death and progression of neurodegenerative processes in many CNS disorders, including multiple sclerosis, Alzheimer’s disease, Parkinson’s disease, and traumatic injury [86,87,88,89]. Thus, preservation of axons and inhibition of neural death are important steps in designing novel therapeutics for treatment of neurodegenerative disease. Studies suggest that the potential for axon re-growth in the adult CNS has been underestimated and may offer new avenues for restoration of neural growth. One idea for designing better neuroprotective strategies is to target the earliest pathologic events so that later degenerative consequences can be avoided. Thus, regulation of NSE may be critical in controlling inflammatory and degenerative processes in SCI and other CNS injury. 

## 6. Conclusions

NSE is a valuable biomarker in neural degeneration and regeneration in SCI and many other CNS disorders. After injury, NSE expression and activity are markedly upregulated in glial and neuronal cells, indicating the enzyme’s role in inflammation following these events. However, NSE also has neurotrophic properties on a broad spectrum of CNS neurons and is required for neuronal cell survival. It is likely that regulation of inflammatory cytokines and chemokines as well as proteases may overcome axonal damage and neuronal death. Cat X can regulate NSE and neuronal survival. Thus, regulation of NSE via Cat X or other avenues may be important therapeutic strategies for prevention of inflammation and neurodegeneration in SCI and many other neurodegenerative diseases. Future studies should focus on the regulation of NSE for optimum attenuation of neuro-inflammation and promotion of neuroprotection in neurodegenerative conditions.
